# Herpes Simplex Virus Type 2 Blocks IFN-β Production through the Viral UL24 N-Terminal Domain-Mediated Inhibition of IRF-3 Phosphorylation

**DOI:** 10.3390/v16101601

**Published:** 2024-10-11

**Authors:** Binman Zhang, Yuncheng Li, Ping Yang, Siyu He, Weilin Li, Miaomiao Li, Qinxue Hu, Mudan Zhang

**Affiliations:** 1 State Key Laboratory of Virology, Wuhan Institute of Virology, Center for Biosafety Mega-Science, Chinese Academy of Sciences, Wuhan 430071, China; zbm321@139.com (B.Z.); liyunc97@163.com (Y.L.); yangping@wh.iov.cn (P.Y.); siyu124@139.com (S.H.); liwl7812@163.com (W.L.); limm0110@163.com (M.L.); 2 Savaid Medical School, University of Chinese Academy of Sciences, Beijing 100049, China

**Keywords:** HSV-2, UL24, IFN-β, IRF-3, immune evasion

## Abstract

Herpes simplex virus type 2 (HSV-2) is a sexually transmitted virus, the cause of genital herpes, and its infection can increase the risk of HIV-1 infection. After initial infection, HSV-2 can establish lifelong latency within the nervous system, which is likely associated with the virus-mediated immune evasion. In this study, we found that HSV-2 UL24 significantly inhibited the activation of the IFN-β promoter and the production of IFN-β at both mRNA and protein levels. Of importance, the inhibitory effect of HSV-2 on IFN-β production was significantly impaired in the context of HSV-2 infection when UL24 was knocked down. Additional studies revealed that, although the full-length HSV-2 UL24 affected cell cycle and viability to some extent, its N-terminal 1–202AA domain showed no obvious cytotoxicity while its C-terminal 201–281 AA domain had a minimal impact on cell viability. Further studies showed that the N-terminal 1–202 AA domain of HSV-2 UL24 (HSV-2 UL24-N) was the main functional region responsible for the inhibition of IFN-β production mediated by HSV-2 UL24. This domain significantly suppressed the activity of RIG-IN, MAVS, TBK-1, IKK-ε, or the IRF-3/5D-activated IFN-β promoter. Mechanistically, HSV-2 UL24-N suppressed IRF-3 phosphorylation, resulting in the inhibition of IFN-β production. The findings of this study highlight the significance of HSV-2 UL24 in inhibiting IFN-β production, revealing two potential roles of UL24 during HSV-2 infection: facilitating immune evasion and inducing cell cycle arrest.

## 1. Introduction

Innate immunity is the first line of host defense against viral infection. The host cells recognize viral components through pattern recognition receptors (PRRs) to clear the invading viruses after viral infection [1,2,3,4], leading to the production of type I interferons (IFNs) and proinflammatory cytokines and the subsequent activation of adaptive immunity [5]. Given that nucleic acids are central to the replication and propagation of most if not all pathogens, the detection of aberrant DNA and RNA has evolved as a fundamental mechanism of host defense against viral infection [6,7,8,9]. The host PRR retinoic acid inducible gene-I (RIG-I) and melanoma differentiation gene-5 (MDA-5) are responsible for recognizing foreign RNA and can be used to detect RNA viruses [9,10,11].

Herpes simplex virus type 2 (HSV-2) is a large dsDNA virus belonging to the subfamily of Herpesviridae [12], which is predominantly sexually transmitted, resulting in recurrent genital herpes [13,14,15,16,17,18]. Both incident and prevalent HSV-2 infections are associated with an increased risk of human immunodeficiency virus type 1 (HIV-1) acquisition [19,20]. As of 2016, an estimated 491.5 million people were infected with HSV-2 worldwide [21]. HSV-2 infection can be divided into acute infection, latent infection, and recurrent infection. The initial infection is at the mucosal site, while HSV-2 migrates to the dorsal root ganglion along the nerve axon to establish latency after the initial infection. HSV-2 is periodically reactivated and then transferred from the dorsal root ganglion to the mucosal lesion [22]. Considering that type I IFNs are essential for mounting a robust host response against viral infection [23,24] and that there are very low levels of IFN-β and IFN-α in the lesional tissue of patient with recurrent mucocutaneous HSV-2 infection [25], it is important to understand how HSV-2 blocks the production of type I IFNs, contributing to its immune evasion of host innate immunity.

Previous studies on herpes simplex virus type 1 (HSV-1) suggested that HSV-1 has evolved complex strategies to block host antiviral immune responses. For instance, the serine/threonine protein kinase US3 of HSV-1 can inhibit type I IFN signals by hyperphosphorylating interferon regulatory factor 3 (IRF-3) and p65 [26,27]. HSV-1 VP24 disrupts the interaction of TANK-binding kinase 1 (TBK-1) with IRF-3 to prevent the activation of IRF-3 and type I IFN production [28]. HSV-1 UL42 interacts with p65 and p50, resulting in the blockade of NF-κB activation [29]. HSV-1 ICP0 directs the proteasomal degradation of several cellular targets as an E3 ubiquitin ligase, thereby interfering with various innate immune responses [30]. HSV-1 ICP47 down-regulates the expression of MHC class I by inhibiting TAP, enabling the virus to evade detection by CD8^+^ T lymphocytes [31]. Although HSV-1 and HSV-2 have a high level of identity in their genomes [32], the function of their viral proteins is not entirely the same. Our previous studies revealed that HSV-2 ICP22 markedly suppressed IFN-β induction, whereas HSV-1 ICP22 did not exhibit such an inhibitory effect [33]. We also found that HSV-2 ICP22 can function as an E3 ubiquitin-protein ligase to inhibit the production of IFN-stimulated genes (ISGs), whereas HSV-1 ICP22 does not possess this activity [34]. Additionally, we observed the role of HSV-2 ICP27 in inhibiting IFN-β production [35]. Given the complexity of the HSV-2 genome encoding more than 70 viral proteins, the involvement of other HSV-2 proteins in viral immune evasion remains to be determined.

In our previous studies, we screened the proteins of HSV-2 that regulate IFN-β expression and found that HSV-2 UL24 down-regulates the activation of the IFN-β promoter. It is known that HSV-1 UL24, as a late gene-encoded multifunctional protein in viral infection, affects several aspects: viral cell-to-cell-spread [36,37,38], accumulation of viral transcripts [39], activation of NF-κB [40], cell cycle [41], dispersal of nucleolin [36], and sub-cellular distribution of viral glycoproteins involved in fusion [42]. However, the functions of HSV-2 UL24 remain largely undefined.

In the current study, we identified the involvement of HSV-2 UL24 in inhibiting IFN-β production. Additional studies revealed that, while the full-length HSV-2 UL24 appears to play a role by affecting cell cycle and viability, its N-terminal 1–202AA domain (HSV-2 UL24-N) is the main functional region in inhibiting IFN-β production without inducing obvious cytotoxicity. We further demonstrated that HSV-2 UL24-N inhibits IFN-β production by blocking IRF-3 phosphorylation.

## 2. Materials and Methods

### 2.1. Viruses, Cell Lines, and Abs

HSV-2 (G strain) was obtained from LGC standards. HSV-1 (F strain) was kindly provided by Professor Chunfu Zheng (University of Calgary). Both HSV-2 and HSV-1 were propagated in African green monkey kidney cells (Vero cells) and titrated by plaque assay on confluent Vero monolayers [43]. Virus stock was supplemented with 10% FBS (Gibico, 1099-141, New York, NY, USA) and stored at −80 °C.

Sendai virus (SeV) was propagated in specific pathogen-free embryonic eggs at 12 days of age. Hemagglutination (HA) assay was performed as previously described using chicken red blood cells [33]. Briefly, embryonated eggs (Beijing Merial Vital Laboratory Animal Technology Corporation, Beijing, China) were incubated at 37 °C for 12 d and then injected with 300 μL SeV at a concentration of 100 hemagglutinating unit (HAU) mL^−1^. After incubation at 37 °C for 72 h and at 4 °C overnight, the allantoic fluids were collected, centrifuged, and then stored at −80 °C. In the HA assay, 2-fold serial dilutions of SeV were prepared and mixed with equal volumes of 1% (*v*/*v*) chicken red blood cells. The mixture was added to 96-well V-shaped bottom plates at room temperature for 45 min. The highest dilution of virus that formed a diffuse lattice was defined as one hemagglutinating unit (HAU).

Human cervical epithelial cell line HeLa, African green monkey kidney cell line Vero, and human embryonic kidney 293T cells (HEK 293T) were cultured in DMEM (Life Technologies, C11995500BT, Carlsbad, CA, USA) supplemented with 10% FBS, 100 U/mL penicillin, and 100 U/mL streptomycin at 37 °C in a 5% CO_2_ incubator.

Ab against IRF-3 was purchased from Proteintech (11312-1-AP, Wuhan, China). Ab against p-IRF-3 was purchased from Cell Signaling Technology (4947S, Boston, MA, USA). Ab against HA tag was purchased from Santa Cruz Biotechnology (sc-7392, Sata Cruz, CA, USA). Ab against Flag tag was purchased from Sigma-Aldrich (F1804, Saint Louis, MO, USA). Abs against β-actin and PCNA were purchased from Proteintech (66009-1-Ig and 10205-2-AP, Wuhan, China).

### 2.2. Plasmid Constructions

The Flag-tagged plasmid expressing IRF-3/5D, RIG-IN, MAVS, TBK-1, or IKK-ε and the reporter plasmids p125-Luc, PRD(III-I)_4_-Luc, and NF-κB-Luc were described in our previous study [33]. The open reading frames (ORF) of HSV-1 and HSV-2 UL24s were amplified from HSV-1 and HSV-2 genomic DNAs, respectively. An N-terminal HA or Flag tag was introduced into HSV-1 or HSV-2 UL24 (named as HSV-2 UL24-Flag, HSV-2 UL24-HA, and HSV-1 UL24-HA) by PCR. PCR products were cloned into pcDNA3.1(+) (Invitrogen). Primers used for plasmid construction are listed in Supplementary Materials Table S1. The Flag- or HA-tagged truncation mutants of HSV-2 UL24 (named HSV-2 UL24-N or UL24-C) were synthesized by Wuhan GeneCreate Biological Engineering Corporation (Wuhan, China). All constructs were validated through DNA sequencing (Sunny Biotechnology, Shanghai, China).

### 2.3. UL24 Knockdown

HSV-2 UL24 siRNA sequences are listed in the Supplementary Materials Table S2. All siRNAs were synthesized by Guangzhou RiboBio Corporation (Guangzhou, China). HeLa cells in 6-well plates were transfected with 200 nM HSV-2 UL24 siRNA or negative control siRNA (N) using Lipofectamine 3000 Reagent (Invitrogen, L3000001, Carlsbad, CA, USA) according to the manufacturer’s instructions. After 10 h of transfection, cells were transfected with 2 μg HSV-2 UL24 or empty vector using X-tremeGENE HP DNA Transfection Reagent (Roche, 6366236001, Basel, Switzerland) according to the manufacturer’s instructions. At 46 h post transfection of HSV-2 UL24 siRNA, cells in 6-well plates were harvested for Western blot assay.

### 2.4. Cell Viability Assay

HEK 293T cells were seeded in 24-well plates for 16 h and then transfected with 500 ng empty vector or plasmid expressing HSV-1 UL24, HSV-2 UL24, or HSV-2 UL24 truncation mutants. At 48 h post transfection, cells were harvested and lysed to measure firefly luciferase activity using a CellTiter-Lumi™ Plus II Luminescent Cell Viability Assay Kit (Beyotime, C0057M, Shanghai, China) according to the manufacturer’s instructions. Briefly, cells were equilibrated at room temperature for 10 min prior to analysis, followed by the addition of a luminescent detection reagent. After an incubation at room temperature for 10 min to stabilize the luminescence signal, the cell viability was detected using a multimode plate reader (PerkinElmer, EnSpire, Waltham, MA, USA).

### 2.5. Luciferase Reporter Assay

HEK 293T cells were seeded in 24-well plates for 16 h and then transfected with 500 ng HSV-2 UL24 or empty vector together with 250 ng p125-Luc and empty vector or plasmid expressing RIG-IN (50 ng), MAVS (50 ng), TBK-1 (50 ng), IKK-ε (50 ng), or IRF-3/5D (50 ng) for 48 h. In some cases, HEK 293T cells were seeded in 24-well plates for 16 h and then transfected with 500 ng HSV-2 UL24 truncation mutant, HSV-2 UL24, or empty vector together with 250 ng p125-Luc, PRD(III-I)_4_-Luc, or NF-κB-Luc. At 24 h post transfection, cells were stimulated with or without SeV for 24 h. Transfections were performed using ExFect Transfection Reagent (Vazyme, T101-01, Nanjing, China) according to the manufacturer’s instructions. Cells were harvested and lysed to measure firefly luciferase activities using a Bio-Lite Luciferase Assay System (Vazyme, DD1201-01, Nanjing, China) according to the manufacturer’s instructions.

### 2.6. RNA Extraction, Reverse Transcription, and Real-Time PCR

Total cellular RNA was extracted using the TRIzol^®^ Reagent (Invitrogen, USA) according to the manufacturer’s instructions. The cDNA was then synthesized using HiScript II Q RT SuperMix (Vazyme, R223-01, China). The newly synthesized cDNA was used as a template for the amplification of IFN-β, GAPDH, and HSV-2 UL24 genes. Primer pairs for IFN-β, GAPDH, and HSV-2 UL24 are shown in Supplemental Materials Table S1. GAPDH was used as an internal control. Relative real-time quantitative PCR was performed on a CFX Connect Real-Time PCR System (Bio-Rad, Hercules, CA, USA) using ChamQ SYBR qPCR Master Mix (High ROX Premixed) (Vazyme, Q341-02, Nanjing, China) according to the following conditions: 95 °C for 30 s, followed by 40 cycles of 95 °C for 10 s and 60 °C for 30 s. The differential gene expression was calculated on the basis of 2^−ΔΔCt^ values. The primers utilized in this study are detailed in Supplemental Materials Table S1.

### 2.7. ELISA for IFN-β

HEK 293T cells, seeded in 6-well plates, were transfected with 2 μg empty vector or plasmid expressing HSV-2 UL24 or its truncation mutant. At 24 h post transfection, cells were stimulated with or without 100 HAU mL^−1^ SeV for 24 h. In certain experiments, HeLa cells in 6-well plates were transfected with 200 nM HSV-2 UL24 siRNA or negative control siRNA (N) for 10 h, followed by infection with HSV-2 at an MOI of 0.1 or mock-infection. At 20 h post infection, cells were stimulated with or without 100 HAU mL^−1^ SeV for 16 h. Cell culture supernatants were collected and centrifuged to remove cell debris. ELISA was carried out to quantify secreted IFN-β. Then, 50 µL of supernatants were used for IFN-β detection with a human IFN-β ELISA kit (R&D PBL, 41410-1, Emeryville, CA, USA) following the manufacturer’s instructions.

### 2.8. Western Blot Analysis

Western blot analysis was carried out as described previously [33]. In brief, cells were lysed with WB/IP lysis buffer (Beyotime, P0013, Shanghai, China) containing protease inhibitor cocktail (Roche, 11697498001, Basel, Switzerland). Nuclear proteins were isolated using a Nuclear-Cytosol Extraction Kit (Applygen, P1200, Beijing, China). Cell extracts were subjected to 10% SDS-PAGE and transferred to PVDF membranes (Millipore 0.45 μm, Boston, MA, USA) followed by blocking with 5% non-fat milk in Tris-buffered saline-Tween (TBST) at room temperature for 2 h. The membrane was cut according to the molecular weight of the protein and incubated overnight with primary antibody at 4 °C. After being washed three times with TBST, the membrane was incubated with HRP-conjugated Goat anti-Rabbit IgG (H + L) (Proteintech, SA00001-2, Wuhan, China) or Goat anti-Mouse IgG (H + L) (Proteintech, SA00001-1, Wuhan, China) at room temperature for 1 h. After being washed three times with TBST, protein bands were visualized by exposure to ChemiScope System (Clinx, Shanghai, China) after the addition of chemiluminescent substrate (Super ECL Plus, S6009M, US EVERBRIGHT, Shanghai, China; Westernbright ECL, K-12045-D50, Advansta, San Jose, CA, USA).

### 2.9. Flow Cytometry Analysis (FCA)

HEK 293T cells, seeded in 6-well plates, were transfected with 2 μg empty vector or plasmid expressing HSV-1 UL24, HSV-2 UL24, or HSV-2 UL24 truncation mutant. At 48 h post transfection, cells were detected using a Cell Cycle and Apoptosis Analysis Kit (Beyotime, C1052, Shanghai, China) according to the manufacturer’s instructions. Briefly, HEK 293T cells were collected and washed with 1× PBS, and then fixed with 70% ethanol overnight at 4 °C. After fixation, cells were washed once with 1× PBS and then resuspended in the assay buffer by addition of RNase A and Propidium Iodide (PI) for 30 min at 37 °C, to analyze the cell cycle by flow cytometry (BD, Fortessa, Franklin, NJ, USA).

### 2.10. Statistical Analysis

All experiments were repeated, and the data are presented as mean ± S.D. with each condition performed in triplicate unless otherwise specified. Data analyses were performed using GraphPad Prism 8 software (GraphPad, San Diego, CA, USA). The comparison between two groups was analyzed using a two-tailed unpaired Student’s *t*-test, while the comparison between more than two groups was analyzed using a one-way ANOVA and Dunnett’s Multiple Comparison Test. The relative intensities of Western blots were quantified using Image J. *p* < 0.05 was considered statistically significant.

## 3. Results

### 3.1. HSV-2 UL24 Inhibits IFN-β Production

**Figure 1:**
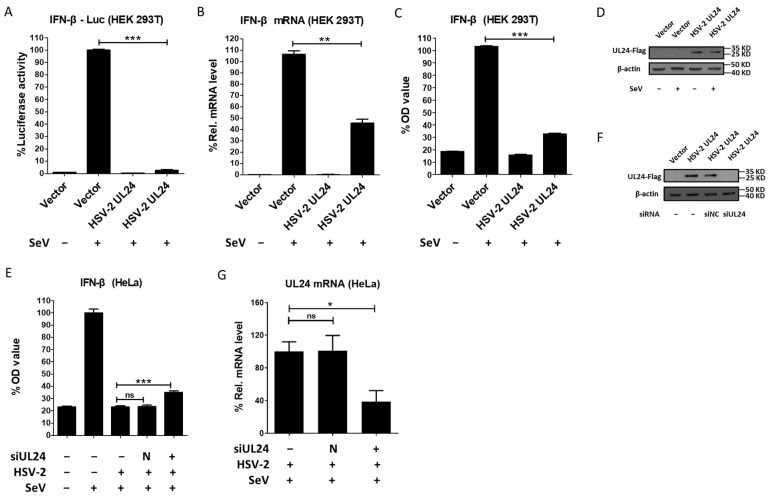
HSV-2 UL24 inhibits IFN-β production. (**A**) HSV-2 inhibits the activation of the IFN-β promoter. HEK 293T cells in 24-well plates were transfected with 500 ng HSV-2 UL24 or empty vector together with 250 ng p125-Luc. At 24 h post transfection, cells were stimulated with or without 100 HAU mL^−1^ SeV for 24 h. (**B**) HSV-2 inhibits the production of IFN-β mRNA. HEK 293T cells in 6-well plates were transfected with 2 μg empty vector or plasmid expressing HSV-2 UL24 for 24 h, followed by stimulation with 100 HAU mL^−1^ SeV for 24 h. (**C**) HSV-2 UL24 inhibits the production of IFN-β. HEK 293T cells in 6-well plates were transfected with 2 μg empty vector or HSV-2 UL24 expression plasmid for 24 h, followed by stimulation with or without 100 HAU mL^−1^ SeV for 24 h. (**D**) The expression of HSV-2 UL24 was detected by Western blot using anti-Flag Ab. (**E**) Knockdown of HSV-2 UL24 impairs the capability of HSV-2 in inhibiting the production of IFN-β. HeLa cells in 6-well plates were transfected with 200 nM HSV-2 UL24 siRNA (siUL24) or negative control siRNA (N). At 10 h post transfection, cells were mock-infected or infected with HSV-2 at an MOI of 0.1. At 20 h post infection, cells were stimulated with or without 100 HAU mL^−1^ SeV for 16 h, and the supernatants were harvested for ELISA. (**F**,**G**) The knockdown effect of HSV-2 UL24 siRNA. HeLa cells in 6-well plates were transfected with 200 nM HSV-2 UL24 siRNA (siUL24) or negative control siRNA (siNC) for 10 h, followed by transfection with 2 μg HSV-2 UL24 expression plasmid or empty vector. At 46 h post transfection of HSV-2 UL24 siRNA, cells were harvested for Western blot assay (**F**). HeLa cells in 6-well plates were transfected with 200 nM HSV-2 UL24 siRNA (siUL24) or negative control siRNA (N). At 10 h post transfection, cells were mock-infected or infected with HSV-2 at an MOI of 0.1. At 20 h post infection, cells were stimulated with or without 100 HAU mL^−1^ SeV for 16 h, and the RNAs were exacted and detected by qPCR (**G**). Values for the samples were expressed as a percentage of the value induced in cells transfected with empty vector or mock-infected with DMEM (**A**–**C**,**E**,**G**). The data shown are representative of three independent experiments, with each condition performed in triplicate (mean ± SD). * *p* < 0.05, ** *p* < 0.01, *** *p* < 0.001. ns, not significantly. N, negative. Rel, relative.

HSV-2 can inhibit type I IFN production [25], but the underlying mechanisms remain elusive. Our previous studies demonstrated that HSV-2 immediate early proteins ICP22 and ICP27 significantly inhibit IFN-β production. However, knockout of ICP22 or knockdown of ICP27 did not fully abolish the inhibitory activity of HSV-2 on IFN-β production in the context of HSV-2 infection [33,35], indicating that other viral proteins are also likely involved in the regulation of IFN-β production. Given the important roles of HSV-1 UL24 [36,37,38,39,40,41,42,44], we investigated whether the late gene UL24 of HSV-2 inhibits IFN-β induction. HEK 293T cells were transfected with an empty vector or plasmid expressing HSV-2 UL24, together with p125-Luc. At 24 h post transfection, cells were stimulated with or without 100 HAU mL^−1^ SeV for 24 h. As shown in Figure 1A, HSV-2 UL24 significantly inhibited the activation of the IFN-β promoter induced by SeV. We further confirmed the effect of HSV-2 UL24 on IFN-β induction at the mRNA level. As shown in Figure 1B, HSV-2 UL24 significantly inhibited the production of IFN-β mRNA. Meanwhile, we detected whether HSV-2 UL24 inhibits the production of IFN-β at the protein level. HEK 293T cells were transfected with an empty vector or plasmid expressing HSV-2 UL24. At 24 h post transfection, cells were stimulated with or without 100 HAU mL^−1^ SeV for 24 h. The level of IFN-β in the supernatants was measured by ELISA, indicating that HSV-2 UL24 significantly inhibited the production of IFN-β at protein level (Figure 1C). The expression of HSV-2 UL24 was confirmed as shown in Figure 1D.

To further confirm the above findings, we next detected whether HSV-2 UL24 could inhibit IFN-β production in the context of virus infection. Given that human cervical epithelial cells are the main target cells of HSV-2 in primary mucosal infection [45], we performed the viral infection assay using the human cervical epithelial cell line HeLa cells. HeLa cells in 6-well plates were transfected with negative control siRNA or HSV-2 UL24-specific siRNA for 10 h, followed by infection with HSV-2 for 20 h. Cells were subsequently stimulated with or without 100 HAU mL^−1^ SeV for 16 h. Our results indicated that the inhibitory effect of HSV-2 on IFN-β production was significantly impaired in the context of HSV-2 infection, with HSV-2 UL24 being knocked down (Figure 1E). The knockdown effect of HSV-2 UL24 siRNA was verified by Western blot (Figure 1F) and qPCR (Figure 1G). Taken together, these results indicate that HSV-2 UL24 inhibits IFN-β production.

### 3.2. The N-Terminal 1–202 AA Domain of HSV-2 UL24 Is the Main Functional Region Responsible for HSV-2 UL24–Mediated Inhibition of IFN-β Production

**Figure 2:**
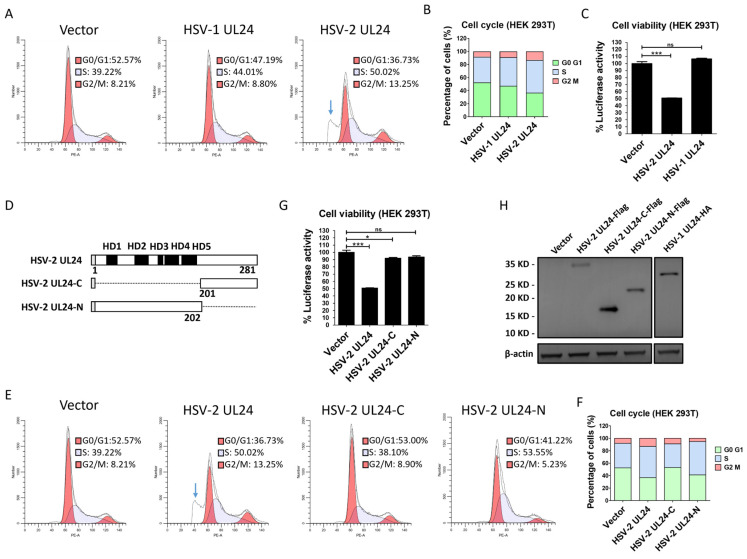
HSV-2 UL24 truncation mutants show no obvious cytotoxicity. (**A**,**B**) HSV-2 UL24 induces cell cycle arrest and cell apoptosis. (**C**) HSV-2 UL24 affects cell viability. (**D**) Schematic representation of HSV-2 UL24 truncation mutants. (**E**,**F**) HSV-2 UL24-N has no obvious cytotoxicity. (**G**) HSV-2 UL24-N has no obvious impact on cell viability. HEK 293T cells in 6-well plates were transfected with 2 μg plasmid expressing HSV-1 UL24 (**A**,**C**), HSV-2 UL24 (**A**,**C**,**E**,**G**,**H**), HSV-2 UL24 truncation mutant (**E**,**G**,**H**) or empty vector. HSV-1 UL24 (**A**,**C**) was used as a control for the arrest of cell cycle. At 48 h post transfection, cells were harvested and stained using a Cell Cycle and Apoptosis Analysis Kit for flow cytometry analysis (**A**,**E**) or lysed using a CellTiter-Lumi™ Plus II Luminescent Cell Viability Assay Kit for luciferase activity analysis (**C**,**G**). The results of A and E were displayed using the stacked bar chart (**B**,**F**). (**H**) The expression of HSV-1 and HSV-2 UL24s and HSV-2 UL24 truncation mutants was detected using anti-Flag or anti-HA Ab. A and E were presented separately, although both were part of the same experiment. Values for the samples were expressed as a percentage of the value in cells transfected with empty vector (**B**,**E**). The data shown are representative of three independent experiments, with each condition performed in triplicate (mean ± SD). * *p* < 0.05, *** *p* < 0.001. ns, not significantly. Blue arrow indicates the apoptotic peak.

Previous studies have shown that HSV-1 UL24 induces cell cycle arrest at the G2 phase [41], while it remains unknown whether HSV-2 UL24 also impacts cell cycle. Of importance, the cell cycle arrest likely leads to the inhibition of IFN-β production. To further confirm the inhibitory effect of HSV-2 UL24 on IFN-β production, we next assessed cell cycle and viability after transfection with HSV-2 UL24 or HSV-1 UL24 using a Cell Cycle and Apoptosis Analysis Kit and a CellTiter-Lumi™ Plus II Luminescent Cell Viability Assay Kit, respectively. The results showed that HSV-2 UL24 not only induced cell cycle arrest at the S/G2 phase (Figure 2A,B), but also had some impact on cell viability (Figure 2C). Additionally, an apoptotic peak was detected after transfection with HSV-2 UL24 (indicated by blue arrow). Of interest, HSV-1 UL24 had little or no impact on the cell cycle or viability compared to HSV-2 UL24. Therefore, we asked whether HSV-2 UL24-mediated inhibition of IFN-β production was due to its cytotoxic capacity. It is known that the HSV-2 UL24 gene is expressed during the late stage of infection, encoding 281 amino acids [46]. Five clusters of highly conserved stretches of amino acids have been identified within the N-terminal portion of UL24, termed the UL24 homology domain (HD) [47,48]. We subsequently constructed two truncation mutants of HSV-2 UL24 by retaining the HD domains (the N-terminal 1–202AA domain, named HSV-2 UL24-N) or removing the HD domains (the C-terminal 201–281 AA domain, named HSV-2 UL24-C), as shown in Figure 2D. The results indicated that HSV-2 UL24-N did not induce an apoptotic peak (Figure 2E) or affect cell viability (Figure 2G), suggesting that HSV-2 UL24-N had no significant cytotoxicity, although it did impact the arrest of the cell cycle at the S phase (Figure 2E). Compared to HSV-2 UL24-N, HSV-2 UL24-C had no impact on cell cycle arrest (Figure 2E) but demonstrated a measurable impact on cytotoxicity, although the effect was minimal compared to that of full-length UL24. The expression of plasmids was confirmed, as shown in Figure 2H. These results collectively demonstrate that the full-length HSV-2 UL24 could impact the cell cycle and viability to some extent, whereas its truncation mutant HSV-2 UL24-N shows no obvious cytotoxicity and HSV-2 UL24-C has a minimal impact on cell viability.

**Figure 3:**
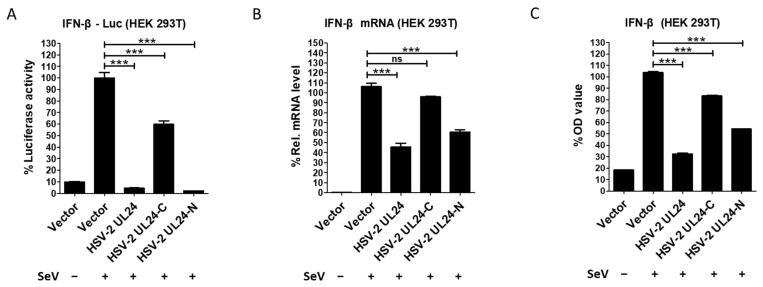
The N-terminal 1–202 AA domain of HSV-2 UL24 is the main functional region responsible for HSV-2 UL24-mediated inhibition of IFN-β production. (**A**) HSV-2 UL24-N inhibits the activation of the IFN-β promoter. HEK 293T cells in 24-well plates were transfected with 500 ng empty vector or plasmid expressing HSV-2 UL24 or its truncation mutant together with 250 ng p125-Luc. At 24 h post transfection, cells were stimulated with or without 100 HAU mL^−1^ SeV for 24 h. (**B**,**C**) HSV-2 UL24-N inhibits the production of IFN-β. HEK 293T cells in 6-well plates were transfected with 2 μg empty vector or plasmid expressing HSV-2 UL24 or its truncation mutant for 24 h, followed by stimulation with or without 100 HAU mL^−1^ SeV for 24 h. The mRNA and protein levels of IFN-β in cells were measured by qPCR (**B**) and ELISA (**C**). Values for the samples were expressed as a percentage of the value induced in cells transfected with empty vector. The data shown are representative of three independent experiments, with each condition performed in triplicate (mean ± SD). *** *p* < 0.001. ns, not significantly. Rel, relative.

Since HSV-2 UL24 but not its truncation mutants showed obvious cytotoxicity, we next examined the inhibitory effect of HSV-2 UL24 on IFN-β production using its truncation mutants. HEK 293T cells were cotransfected with the reporter plasmid p125-Luc and an empty vector or plasmid expressing HSV-2 UL24 truncation mutant for 24 h, followed by stimulation with or without 100 HAU mL^−1^ SeV for 24 h. The results showed that HSV-2 UL24-C had a limited inhibitory effect on the activation of the IFN-β promoter, whereas HSV-2 UL24-N significantly blocked the activation of the IFN-β promoter, similar to the full-length UL24. This suggests that UL24-N is more potent than UL24-C as the functional domain of UL24 for this inhibitory effect (Figure 3A). We also confirmed the inhibitory effect of HSV-2 UL24 truncation mutants on IFN-β production at both mRNA and protein levels. In agreement with the results at the promoter level, HSV-2 UL24-N significantly downregulated the production of IFN-β at both mRNA (Figure 3B) and protein (Figure 3C) levels, while HSV-2 UL24-C had a minimal inhibitory effect. These results together indicate that, while the full-length HSV-2 UL24 can induce cell cycle arrest and cell apoptosis, the N-terminal 1–202 AA domain of HSV-2 UL24 is the main functional region for HSV-2 UL24-mediated inhibition of IFN-β production.

### 3.3. The N-Terminal 1–202 AA Domain of HSV-2 UL24 Interferes with the IRF-3-Mediated Signaling Pathway

**Figure 4:**
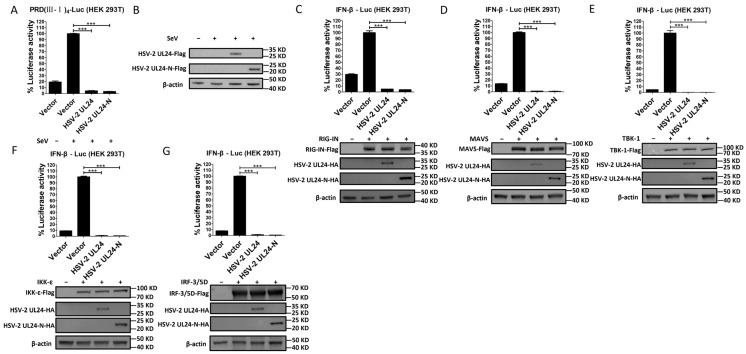
The N-terminal 1–202 AA domain of HSV-2 UL24 interferes with the IRF-3-mediated signaling pathway. (**A**) HSV-2 UL24-N inhibits the activation of the IRF-3-responsive IFN-β promoter. HEK 293T cells in 24-well plates were transfected with 500 ng empty vector or plasmid expressing HSV-2 UL24 or UL24 truncation mutant together with 250 ng PRD(III-I)_4_–Luc. At 24 h post transfection, cells were stimulated with or without HAU mL^−1^ SeV for 24 h. The expression of HSV-2 UL24 and UL24-N was detected by Western blot using anti-Flag Ab (**B**). (**C**–**G**) HSV-2 UL24-N inhibits the activation of the IRF-3 signaling pathway. HEK 293T cells in 24-well plates were cotransfected with 500 ng empty vector or plasmid expressing HSV-2 UL24 or its truncation mutant together with 250 ng p125-Luc and 50 ng plasmid expressing RIG-IN (**C**), MAVS (**D**), TBK-1 (**E**), IKK-ε (**F**), or IRF-3/5D (**G**). At 48 h post transfection, cells were harvested and luciferase activities were measured. Values for the samples were expressed as a percentage of the value induced in cells transfected with empty vector (**A**, **C**–**G**). Protein expression was detected by Western blot using anti-HA or anti-Flag Ab. The data shown are representative of three independent experiments, with each condition performed in triplicate (mean ± SD). *** *p* < 0.001.

The IRF-3-mediated signaling pathway is well known for its critical role in IFN-β induction [49,50]. To gain insights into the potential mechanism of the HSV-2 UL24-mediated blockade of IFN-β production, we first investigated whether HSV-2 UL24-N interferes with the IRF-3-mediated signaling pathway. HEK 293T cells were cotransfected with plasmid expressing HSV-2 UL24-N and the reporter plasmid PRD(III-I)_4_-Luc, which contains four repeats of the IRF-3 responsive domain of the IFN-β promoter. At 24 h post transfection, cells were stimulated with or without 100 HAU mL^−1^ SeV for 24 h. As shown in Figure 4A, B, HSV-2 UL24-N significantly inhibited the activation of the IRF-3-responsive promoter induced by SeV, indicating that HSV-2 UL24-N inhibits IFN-β production, likely through the IRF-3-dependent signaling pathway.

To further confirm whether HSV-2 UL24-N directly affects the IRF-3 signaling pathway, plasmid-expressing RIG-IN, MAVS, TBK-1, IKK-ε, or IRF-3/5D, which are inducers of IFN-β in the IRF-3 signaling pathway, was transfected into HEK 293T cells together with p125-Luc and HSV-2 UL24 or UL24-N expression plasmid or empty vector. As shown in Figure 4C–G, in the presence of HSV-2 UL24 or UL24-N, the activation of the IRF-3 pathway by the above components was abolished. These results indicate that the N-terminal 1–202 AA domain of HSV-2 UL24 interferes with the IRF-3-mediated signaling pathway by acting on the downstream of IRF-3.

### 3.4. The N-Terminal 1–202 AA Domain of HSV-2 UL24 Inhibits the Phosphorylation of IRF-3

**Figure 5:**
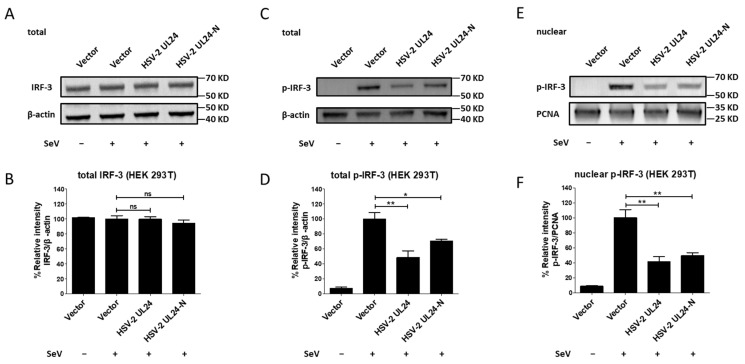
The N-terminal 1-202 AA domain of HSV-2 UL24 inhibits the phosphorylation and nuclear translocation of IRF-3. (**A**,**B**) HSV-2 UL24-N does not affect the expression of IRF-3. (**C**,**D**) HSV-2 UL24-N suppresses the phosphorylation of IRF-3. (**E**,**F**) HSV-2 UL24-N induces the decrease in nuclear translocation of IRF-3. HEK 293T cells in 6-well plates were transfected with 2 μg empty vector or plasmid expressing HSV-2 UL24 or UL24 truncation mutant. At 24 h post transfection, cells were stimulated with or without 100 HAU mL^−1^ SeV for 24 h. Cells were directly lysed to perform Western blot or collected to isolate the nuclear protein. Total cellular IRF-3 (**A**,**B**) and p-IRF-3 (**C**,**D**) or nuclear p-IRF-3 (**E**,**F**) were detected by Western blot using anti-IRF-3 or anti-p-IRF-3 Ab. β-actin and PCNA were used as loading controls. The relative intensities of IRF-3 and p-IRF-3 blots were quantified using Image J (**B**,**D**,**F**). One representative experiment out of three is shown. * *p* < 0.05, ** *p* < 0.01, ns, not significantly.

Given that HSV-2 UL24-N interferes with the IRF-3-mediated signaling pathway by acting on the downstream of IRF-3, we asked whether it affected IRF-3 activation. The activation of IRF-3 involves two major steps: phosphorylation and nuclear translocation [50,51]. Therefore, we next detected whether HSV-2 UL24-N affected the phosphorylation and nuclear translocation of IRF-3. HEK 293T cells were transfected with plasmid expressing HSV-2 UL24, HSV-2 UL24-N, or empty vector for 24 h, followed by stimulation with or without 100 HAU mL^−1^ SeV for 24 h. The results indicated that HSV-2 UL24-N had no effect on the expression level of IRF-3 (Figure 5A,B), but the phosphorylation (Figure 5C,D) and nuclear translocation (Figure 5E,F) of IRF-3 were significantly reduced in HSV-2 UL24-N-transfected cells. Collectively, these results indicate that the N-terminal 1–202 AA domain of HSV-2 UL24 inhibits the phosphorylation of IRF-3, reducing its nuclear translocation and ultimately blocking IRF-3 activation.

## 4. Discussion

Herpes simplex virus type 2 (HSV-2), a large dsDNA virus, is mostly sexually transmitted and can establish a lifelong latent infection [12,14,15,52]. Both incident and prevalent HSV-2 infections are associated with an increased risk of HIV-1 infection [19,20]. Innate immunity is the first line of the body’s defense against foreign pathogens [53,54], with IFN-β being a key immune factor in the immune responses [55]. It is known that HSV-2 lesions contained a large amount of IFN-γ, but a very low amount of type I IFN [25], suggesting that HSV-2 has evolved certain immune evasion mechanisms to facilitate its infection.

We previously reported that HSV-2 immediate early proteins ICP22 and ICP27 inhibited IFN-β production through different mechanisms [33,35]. In the current study, we found that the late protein of HSV-2, UL24, significantly suppressed the RNA virus-induced production of IFN-β. It is generally believed that RNA viruses are recognized by RNA sensors, such as RIG-I and MDA5 [11], while DNA viruses are recognized by DNA sensors, such as cGAS [6]. Given that DNA viruses produce the by-product of dsRNA during their replication cycle [56], which could be recognized by RNA sensors such as RIG-I [9,10,11], understanding the mechanisms underlying how HSV-2 interferes with host RNA sensors would shed light on the complex network of HSV-2 immune evasion.

To further identify the inhibitory effect of HSV-2 UL24 on IFN-β production in the context of HSV-2 infection, we found that the inhibitory capability of HSV-2 on IFN-β production was significantly impaired but not completely abolished when HSV-2 UL24 was knocked down, suggesting the involvement of other viral components in inhibiting IFN-β production, which is in accordance with our previous studies [33,35].

In a previous study, HSV-1 UL24 clearly induced cell cycle arrest at the G2 phase [41]. In the current study, we found that HSV-1 UL24 had little effect on the cell cycle. Compared to HSV-1 UL24, HSV-2 UL24 had a greater impact on cell cycle regulation, as well as cell apoptosis and viability. To understand whether the inhibitory effect of HSV-2 UL24 on IFN-β production was due to its cytotoxic capacity, we constructed two truncation mutants of HSV-2 UL24 (UL24-N and UL24-C). We observed that the truncation mutant UL24-N (the N-terminal 1–202AA domain of HSV-2 UL24), containing five highly conserved homology domains (HDs), had no obvious cytotoxicity, while it could significantly inhibit the production of IFN-β similar to the full-length UL24. Compared to HSV-2 UL24-N, UL24-C had a minimal effect on IFN-β production. These suggest that UL24-N is more potent than UL24-C as the functional domain of UL24 for this inhibitory effect. According to these findings, we infer that HSV-2 UL24 affects cell cycle and viability depending on its complete spatial structure, whereas its inhibitory effect on IFN-β production mainly relies on its N-terminal 1–202AA domain, which contains HD structures. It is known that the N-terminal domain of HSV-1 UL24 (1-192 AA) drives the redistribution of nucleolin and B23, and is important for the pathogenesis of HSV-1 ocular infection [36,37,48,57], while the C-terminal domain of HSV-1 UL24 (190-269 AA) is necessary and sufficient for targeting the Golgi apparatus and influencing the syncytial plaque phenotype [48,58]. The precise roles of the N- and C-terminal domains of HSV-2 UL24 require further exploration in future studies.

To further elucidate the mechanism underlying HSV-2 UL24-mediated inhibition of IFN-β production, various components of the dsRNA-signaling pathway were overexpressed to assess the impact of UL24 expression on the activation of the IFN-β promoter. The results showed that UL24 and UL24-N could effectively counteract the overexpression of these components and even inhibit the constitutive activity of IRF-3/5D. Therefore, we focused on IRF-3 activation and found that HSV-2 UL24-N interferes with the phosphorylation of IRF-3. IRF-3 is expressed in many types of cells, playing a unique and important role in the induction of IFN-β in response to viral infection [50,51,59]. Studies have shown that several viral proteins, including HSV-1 US3 and VP24, and HSV-2 ICP27 regulate IFN-β production by regulating IRF-3 phosphorylation [26,28,35]. Of interest, HSV-1 UL24 was reported to abrogate the DNA-sensing signal pathway via blocking the activation of NF-κB rather than the IRF-3 responsive promoter [40]. In the current study, we found that HSV-1 UL24 also inhibits the activation of the IRF-3 responsive promoter (Supplemental Materials Figure S1A,B). This inconsistence may be attributed to differences in viral strains [60]. Our HSV-1 UL24 was derived from the F strain, whereas the HSV-1 KOS strain was used in the previous study [40]. The UL24 proteins of HSV-1 KOS and F strains differ by three amino acids at positions 31, 226, and 231. While our current study mainly focuses on HSV-2, future studies are needed to identify the roles of these amino acids.

Given that the promoter of IFN-β contains binding sites for both IRF-3 and NF-κB, we detected whether HSV-2 UL24 affects NF-κB activation. As shown in Supplemental Materials Figure S1C, NF-κB activation was indeed affected by HSV-1 UL24, HSV-2 UL24, or its truncation mutant HSV-2 UL24-N, although the effect was much weaker than that on IFN-β. These findings imply that HSV-2 UL24 likely inhibits IFN-β production via both the IRF-3 and NF-κB signaling pathways. Although it extends beyond the scope of this work, expanding on the functional mechanism of UL24 is worthwhile in future studies. In the current study, we found that the N-terminal 1–202 AA domain of HSV-2 UL24 inhibits IRF-3 phosphorylation through the IRF-3 signaling pathway. Furthermore, a potential interaction between UL24 and IRF-3 was simulated using an online platform (https://lab.chaidiscovery.com/ (accessed on 24 September 2024)), revealing that a number of hydrogen bonds existed between UL24 and IRF-3 (see Supplementary Materials Figure S2), although co-immunoprecipitation experiments did not show a clear interaction between UL24 and IRF-3. We infer that the interaction between UL24 and IRF-3 is likely too weak to be effectively pulled down using specific antibodies.

HSV-2 and HSV-1 are highly homologous [32]. Most previous studies have focused on HSV-1 [26,27,28,29,40]. HSV-1 UL24 has been shown to be a multifunctional protein in viral infection. It can affect the viral cell-to-cell-spread [37,38], the accumulation of viral transcripts [39], the dispersal of nucleolin, the cell fusion of HSV-1 [36,42], and the productive infection of mouse sensory ganglia [44]. However, little is known concerning the function of HSV-2 UL24. In this study, we found that HSV-2 UL24, at least in part, likely plays two potential roles: facilitating immune evasion and inducing cell cycle arrest, depending on its different domains during HSV-2 infection. Given that cell apoptosis induced by herpesvirus was implicated to activate viral replication [61], although beyond the scope of this study, future work is warranted to explore the cytotoxic function and structural characteristics of HSV-2 UL24 in relation to its functions in the process.

**Figure 6:**
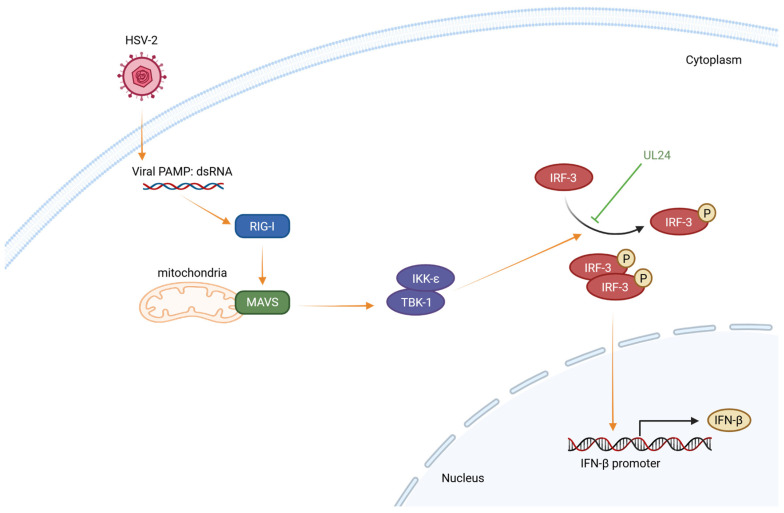
The schematic model illustrates the mechanism by which HSV-2 UL24 blocks the IRF-3 signaling pathway. During HSV-2 infection, the viral dsRNA by-product is recognized by RIG-I. Subsequently, RIG-I binds to dsRNA and signals to the adaptor protein MAVS. Dimerized MAVS recruits and activates the downstream protein kinase complexes TBK-1/IKK-ε, leading to the phosphorylation and dimerization of IRF-3. The IRF-3 dimer translocates from the cytoplasm to the nucleus, where it binds to the IFN-β promoter to initiate the transcription of IFN-β. In the case of HSV-2 infection, the viral late protein UL24 interferes with IRF-3 phosphorylation, leading to the blockade of IFN-β production.

In conclusion, we demonstrate that the N-terminal 1–202 AA domain of HSV-2 UL24 is the main functional region responsible for the HSV-2 UL24-mediated inhibition of IFN-β production by inhibiting IRF-3 phosphorylation. A proposed model is illustrated in Figure 6. During HSV-2 infection, the viral dsRNA by-product is recognized by RIG-I. Subsequently, RIG-I binds to dsRNA and signals to the adaptor protein MAVS [56,62]. Dimerized MAVS recruits and activates the downstream protein kinase complexes TBK-1/IKK-ε, leading to IRF-3 phosphorylation and dimerization [56,63]. The IRF-3 dimer then translocates from the cytoplasm to the nucleus, where it binds to the IFN-β promoter to initiate the transcription of IFN-β [64]. In the case of HSV-2 infection, the viral late protein UL24 interferes with IRF-3 phosphorylation, leading to the blockade of IFN-β production.

## Data Availability

Data are contained within the article.

## Supplementary Materials

The following supporting information can be downloaded at: https://www.mdpi.com/article/10.3390/v16101601/s1, Figure S1: HSV-1 and HSV-2 UL24 interfere with the IRF-3–mediated and NF-κB–mediated activation of IFN-β promoter; Figure S2. The interaction of UL24 with IRF3 was simulated using an online platform; Table S1: The primers used in this study; Table S2: The siRNA used in this study.
