# Supplementary material for: Herpes Simplex Virus Type 2 Blocks IFN-β Production through the Viral UL24 N-Terminal Domain-Mediated Inhibition of IRF-3 Phosphorylation
Source: Viruses. 2024 Oct 11;16(10):1601. doi: 10.3390/v16101601 (PMC11512255; doi:10.3390/v16101601)
Supplement: Supplementary file 1 [file viruses-16-01601-s001.zip › viruses-3114836-supplementary.pdf]

Supplementary Materials

Herpes simplex virus type 2 blocks IFN-β production through the viral UL24 N-terminal domain-mediated inhibition of IRF-3 phosphorylation

Binman Zhang [1, 2], Yuncheng Li [1, 2], Ping Yang [1, 2], Siyu He [1, 2], Weilin Li [1, 2], Miaomiao Li [1, 2], Qinxue Hu [1, 2 *] and Mudan Zhang [1, 2*]

[1] State Key Laboratory of Virology, Wuhan Institute of Virology, Center for Biosafety Mega-Science, Chinese Academy of Sciences, Wuhan, 430071, China;

[2] Savaid Medical School, University of Chinese Academy of Sciences, Beijing, 100049, China;

[*] Correspondence: E-mail: mudan@wh.iov.cn; E-mail: qhu@wh.iov.cn; Tel.: +86-27-87998706 (Q.H. and M.Z.)

Tables

Supplementary Table S1

The primers used in this study.

Gene	Primers (5'→3')		Purpose
HSV-2 UL24-Flag	Forward	CGGATCCATGGATTACAAGGATGACGACGATAAGGCTAGGACGGGACGC	plasmid construction
	Reverse	ATCTCGAGTCACTCGGTTTTGGTCCGGG	
HSV-2 UL24-HA	Forward	CGGGATCCATGTACCCATACGACGTCCCAGACTACGCTGCTAGGACGGGACGC	plasmid construction
	Reverse	ATCTCGAGTCACTCGGTTTTGGTCCGGG	
HSV-1 UL24-HA	Forward	AAGCTTGGTACCGAGCTCGGATCCATGTACCCATACGACGTCCCAGACTACGCTGCCGCGAGAACGCGC	plasmid construction
	Reverse	TTAAACGGGCCCTCTAGACTCGAGTCATTCGGAGGCGGCTCGGG	
GAPDH	Forward	GGGAAGCTCACTGGCATGG	qPCR

	Reverse	TTACTCCTTGGAGGCCATGT	
IFN-β	Forward	CAAATTGCTCTCCTGTTGTGCTTC	qPCR
	Reverse	AATGCGGCGTCCTCCTTCT	
HSV-2 UL24	Forward	CCACCAGGATTTGTGGAACG	qPCR
	Reverse	TGCAGCAGCTTCAGGGAGTG	

Supplementary Table S2

The siRNAs used in this study.

**Gene**	**Target gene (5'→3')**
Negative siRNA	TTCTCCGAACGTGTCACGT
HSV-2 UL24 siRNA	CTAAAGACATGCAAATCGA

Supplementary Figure

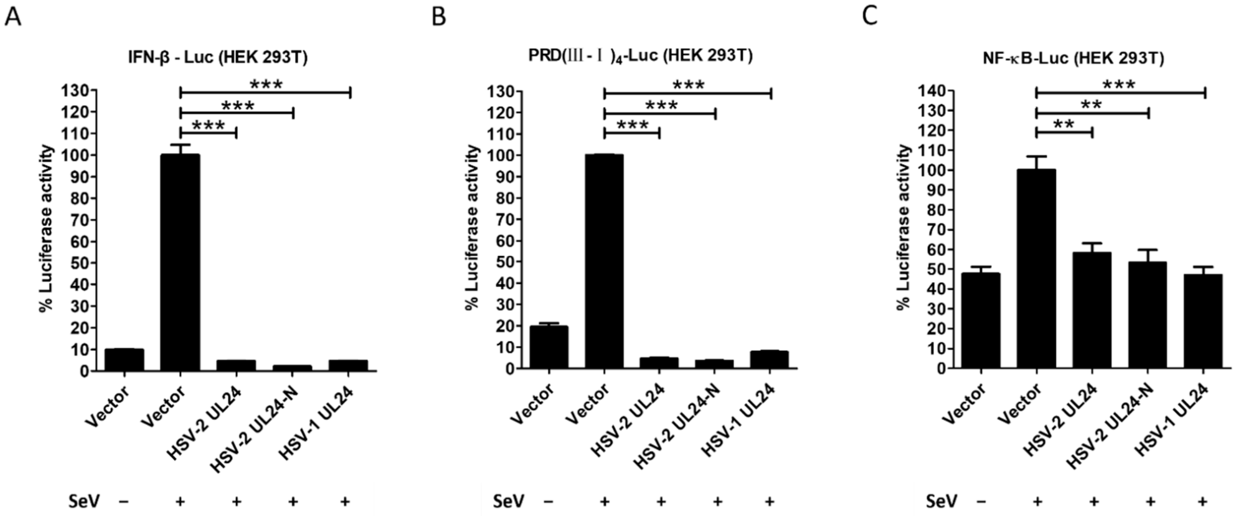

Figure S1. HSV-1 and HSV-2 UL24s interfere with IRF-3– or NF-κB–mediated promoter activation. HEK 293T cells in 24-well plates were transfected with 500 ng plasmid expressing HSV-1 UL24, HSV-2 UL24, HSV-2 UL24 truncation mutant or empty vector together with 250 ng p125-Luc **(A)**, PRD(III-I)4-Luc **(B)**, or NF-κB-Luc **(C)**. At 24 h post transfection, cells were stimulated with or without 100 HAU mL^{-1} SeV for 24 h. The luciferase activity was determined by luciferase reporter assay. Values for the samples were expressed as a percentage of the value induced in cells transfected with the empty vector. Data shown are mean ± S.D. of three independent experiments with each condition performed in triplicate. For image, one representative experiment out of three is shown. $^{**}p < 0.01$, $^{***} p < 0.001$.

A

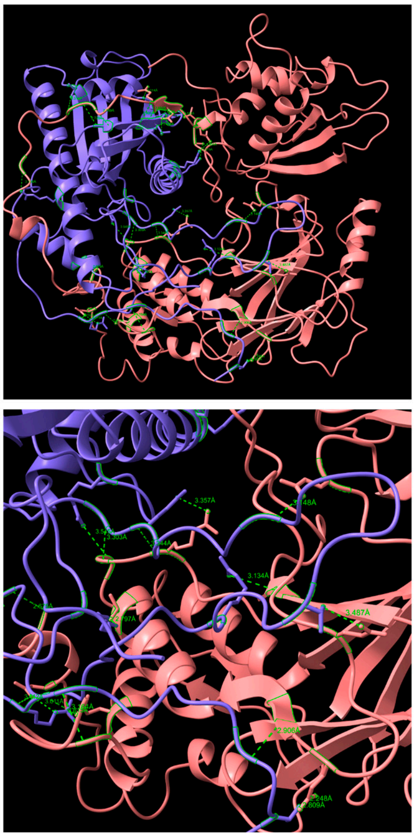

B

reveal true log true

```
Finding intermodel H-bonds
Finding intramodel H-bonds
Constraints relaxed by 0.4 angstroms and 20 degrees
Models used:
        1 pred.model_idx_0.cif

28 H-bonds
H-bonds (donor, acceptor, hydrogen, D..A dist, D-H..A dist):
/A ARG 15 NH2   /B THR 331 OG1   no hydrogen  2.797  N/A
/A ARG 31 NH2   /B ASP 154 O     no hydrogen  3.136  N/A
/A THR 55 OG1   /B VAL 148 O     no hydrogen  3.316  N/A
/A ARG 87 NH2   /B GLY 335 O     no hydrogen  3.545  N/A
/A ARG 137 NH2  /B ASP 138 OD2   no hydrogen  3.404  N/A
/A LYS 141 NZ   /B SER 123 OG    no hydrogen  3.019  N/A
/A VAL 170 N    /B MET 147 O     no hydrogen  2.720  N/A
/A VAL 173 N    /B GLU 142 O     no hydrogen  2.805  N/A
/A THR 174 OG1  /B ASP 141 OD1   no hydrogen  3.403  N/A
/A ARG 175 N    /B LEU 140 O     no hydrogen  2.813  N/A
/A ARG 175 NE   /B GLU 142 OE2   no hydrogen  3.014  N/A
/A ARG 211 N    /B ASP 177 OD1   no hydrogen  3.112  N/A
/A ARG 211 NE   /B ASP 177 OD1   no hydrogen  3.011  N/A
/A ARG 221 NH1  /B VAL 295 O     no hydrogen  3.134  N/A
/A THR 226 OG1  /B GLU 310 OE1   no hydrogen  3.487  N/A
/A GLY 234 N    /B GLN 354 OE1   no hydrogen  3.148  N/A
/A ASP 242 N    /B SER 336 O     no hydrogen  3.303  N/A
/A ALA 271 N    /B GLN 422 O     no hydrogen  3.164  N/A
/A THR 280 OG1  /B GLU 316 OE1   no hydrogen  2.248  N/A
/A THR 280 OG1  /B GLU 316 OE2   no hydrogen  2.809  N/A
/B THR 122 OG1  /A PRO 178 O     no hydrogen  1.583  N/A
/B GLU 142 N    /A VAL 173 O     no hydrogen  2.653  N/A
/B MET 147 N    /A VAL 170 O     no hydrogen  2.769  N/A
/B ARG 172 N    /A LEU 265 O     no hydrogen  2.675  N/A
/B GLY 318 N    /A ARG 277 O     no hydrogen  2.906  N/A
/B ARG 338 N    /A GLU 240 O     no hydrogen  3.344  N/A
/B ARG 338 NH2  /A GLU 240 OE1   no hydrogen  3.357  N/A
/B GLN 413 NE2  /A ARG 212 O     no hydrogen  2.815  N/A
```

28 hydrogen bonds found

Figure S2. The interaction of UL24 with IRF3 was simulated using an online platform (https://lab.chaidiscovery.com/). A. The hydrogen-bonds were highlighted in green between UL24 and IRF-3, while IRF3 and UL24 were indicated in pink and purple, respectively. B. The numbers of hydrogen-bonds between UL24 and IRF3 (A and B indicate UL24 and IRF3, respectively).
